# A Case Study on Vestibular Sensations in Driving Simulators

**DOI:** 10.3390/s22155837

**Published:** 2022-08-04

**Authors:** Jose V. Riera, Sergio Casas, Francisco Alonso, Marcos Fernández

**Affiliations:** 1 Computer Science Department, Higher Technical School of Engineering, University of Valencia, 46010 Valencia, Spain; 2 Institute of Robotics, Information Technologies and Communication Research (IRTIC), University of Valencia, 46010 Valencia, Spain; 3 Faculty of Psychology, University of Valencia, 46010 Valencia, Spain; 4 Research Institute on Traffic and Road Safety (INTRAS), University of Valencia, 46010 Valencia, Spain

**Keywords:** driving simulator, motion platform, motion cueing algorithm, washout filter, car characterization, vestibular perception

## Abstract

Motion platforms have been used in simulators of all types for several decades. Since it is impossible to reproduce the accelerations of a vehicle without limitations through a physically limited system (platform), it is common to use washout filters and motion cueing algorithms (MCA) to select which accelerations are reproduced and which are not. Despite the time that has passed since their development, most of these algorithms still use the classical washout algorithm. In the use of these MCAs, there is always information that is lost and, if that information is important for the purpose of the simulator (the training simulators), the result obtained by the users of that simulator will not be satisfactory. This paper shows a case study where a BMW 325Xi AUT fitted with a sensor, recorded the accelerations produced in all degrees of freedom (DOF) during several runs, and data have been introduced in mathematical simulation software (washout + kinematics + actuator simulation) of a 6DOF motion platform. The input to the system has been qualitatively compared with the output, observing that most of the simulation adequately reflects the input to the system. Still, there are three events where the accelerations are lost. These events are considered by experts to be of vital importance for the outcome of a learning process in the simulator to be adequate.

## 1. Introduction

Road traffic accidents are a global problem with social, economic and public health repercussions [1]. Governmental entities worldwide develop prevention measures and strategies to reduce road accidents and mitigate the devastating effects of this problem [2]. Data indicate that more than 90% of crashes are due to the human factor, as road users commit infractions or engage in risky behaviors with fatal consequences [3]. In this sense, adequate training and education of drivers are essential to achieve a real awareness of the dangers of the road and to provide all the tools necessary to prevent traffic accidents.

In recent decades, technological devices and graphics-based simulators with various functionalities have been developed to meet the needs of users. The first simulators that were created were airplane simulators, developed by the U.S. Army. Since then, all the technologies involved with simulators (graphics, audio, projection, motion, steering wheels, sensing) have evolved in an extraordinary way. It was in the 1960s and 1970s when, with the advent of digital computers, the simulators known as full-motion [4] appeared, which besides recreating a virtual scenario, simulated the accelerations that occurred. As costs decreased, simulators of all types of vehicles began to emerge: flight [5], ship [6,7], car [8], truck [9], and submarine [10], among many others.

Currently, the use of driving simulators is focused on entertainment and training. The common link in both cases is that the simulator designer always tries to achieve the maximum level of abstraction of reality for the user. To achieve this, they must introduce stimuli for as many senses as possible: sight, sound, balance, smell, touch, etc. Scientific evidence indicates that realistic and dynamic training using simulators improves the user’s decision-making, cognitive functions and, consequently, performance and skill in the on-road driving task [11,12,13].

There is currently some controversy [14] in society about the use of driving simulators for training and to obtain validation of the skills of new drivers who opt for a driving license. De Winter [15] analyzes the advantages and disadvantages of using driving simulators. On the one hand, there are those who defend that a simulator can never be compared to real driving [16,17,18]. On the other hand, other authors clearly bet on increasing the use of simulators as training tools [19,20], since this allows them to select the situations that they want to reproduce (rain, snow, a traffic accident, etc.) while providing safety for novice users. In the opinion of the authors, the only way to get the first group to accept the simulators as useful tools for training is to achieve a high level of immersion for the users and, for this, all the sensations perceived must be similar to those experienced in a real vehicle, including of course, the motion cues.

The research in this paper focuses on motion perception cues since the literature indicates that this variable is fundamental to generating a realistic driving sensation, motion being an element placed above other variables such as the state of the cabin or the sound to generate realism to the user [21]. These cues are based on the information provided by the vestibular system. The way to introduce sensations through a simulator is mainly through motion platforms. These have been implemented in simulators of all types [22] and are the source of a large number of publications [23,24,25]. 

Thanks to motion platforms, simulators become more realistic, since it is possible to reproduce accelerations similar to those felt by a user who is actually driving the simulated vehicle (motion characterization [26]). In addition, with the correct configuration of the system, it reduces the possible lack of coordination of the senses, which may result in dizziness or nausea [27].

**Figure 1:**
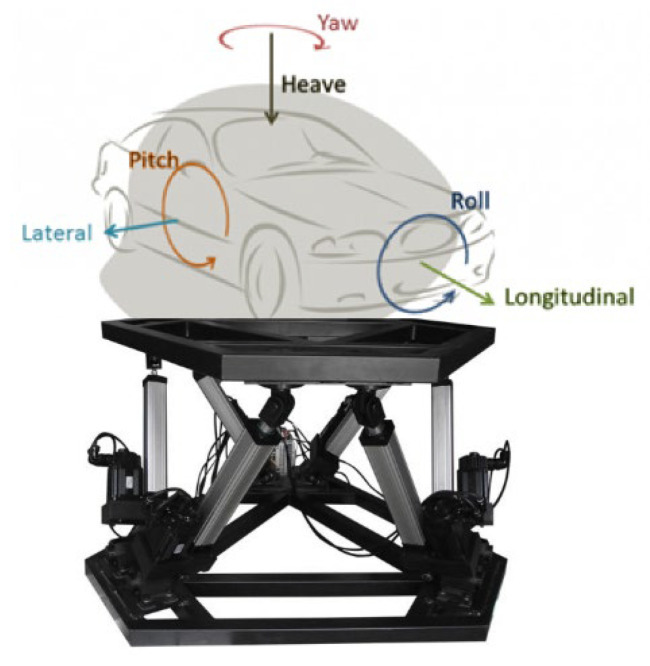
Stewart 6DOF motion platform.

Motion platforms are usually two, three or six degrees of freedom (DOF), although there are prototype platforms with other DOFs [10]. One of the most commonly used platform types with 6DOFs are known as Stewart platforms [28]. Evolved models of these platforms have been made [29], but the most widespread is still the original Stewart platform. The main reason for building platforms that are not six degrees of freedom is cost, since the more degrees of freedom, the more actuators and controllers are needed. Figure 1 shows the existing six degrees of freedom, which are translations and rotations about the three axes.

However, motion platforms have a great challenge, which consists of trying to reproduce the accelerations of a vehicle with no limitation, by means of an actuator system limited in space and in which each DOF is dependent on the other [30,31]. For example, when a car accelerates on a straight road with variable accelerations for 20 consecutive seconds, it produces accelerations that are difficult to recreate with a motion platform with a very limited range of motion in each DOF due to its mechanical construction.

In order to try to solve this problem, there are washout algorithms (or filters) that receive, as input, the accelerations and angular velocities that are being produced in a simulated vehicle and obtain, as a result, the desired positions of each DOF. The set of these positions is sent to a module known as inverse kinematics [32], which is in charge of calculating the position in which each actuator must be placed at each moment to achieve the DOFs requested by the washout. 

There are countless implementations of these algorithms, but the most widespread is the one known as classical washout [33]. One of the most complex parts in the development of a simulator with a motion platform lies in properly configuring the filters used by this type of algorithm. There is ample state of the art documentation in this regard, but concrete details are rarely given on the different configurations used; given the costly process of configuring these filters, companies and research centers are usually reluctant to publish the results. The configuration of these filters will determine which accelerations will be reproduced by the motion platform and which will not. What is certain is that it is difficult to reproduce all of them due to the physical limitations of the platform actuators [34].

Depending on the lost accelerations, there will be details of the simulation produced that will not be perceived by the user. Depending on the use of the simulator, these lost accelerations (and therefore the event that has produced them) will be of greater or lesser relevance. 

**Figure 2:**
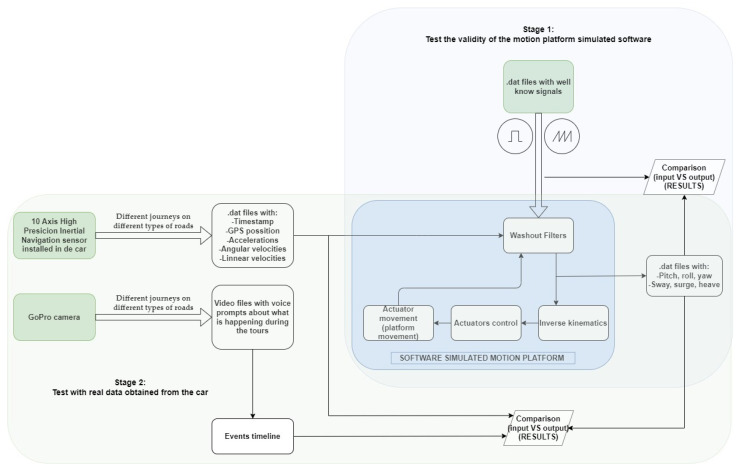
Outline of the tests to be performed.

In the present case, we will try to locate the accelerations that are lost with a standard washout configuration for a car driving training simulator consisting of a 6DOF motion platform. For this purpose, a real car will have sensors installed (accelerometers and gyroscopes) that will provide the real accelerations of the car on different journeys on different types of roads. Specifically, the car is a BMW 325Xi AUT, which is considered a mid-range car. These data will be introduced in software that simulates the behavior of a 6DOF motion platform and all the surrounding software, including the washout filters. Previously, the correct operation of this software will be validated by introducing well-known signals and analyzing the expected results. The scheme can be seen in Figure 2. 

Depending on the results obtained, the importance or not of the lost data will be evaluated, studying if it is possible to implement any solution. The data obtained will be evaluated to locate the possible losses of motion fidelity, locating the action that was being performed in driving (speed, type of road, specific event, etc.) at that particular time and analyzing the importance of this loss depending on the possible uses to be made of a simulator (gaming, training, etc.). Table 1 shows a summary of the advantages and disadvantages of this research.

The document is organized as follows: Section 2 describes the materials and methods used, first describing the data collection in the real car and then describing the tests carried out with the motion platform simulator. Section 3 shows the observed results, highlighting three events in which motion cues are lost in the motion platform simulator. Section 4 shows a discussion about the importance of these events according to the use of the simulator. Finally, Section 5 includes the conclusions and future work.

## 2. Materials and Methods

The following sections show the tests carried out. First, data were collected from a real car. Next, the platform simulation software was checked for correct operation by entering well-known signals, and finally, the accelerations collected in the first phase were entered into the software to observe the results.

### 2.1. Real Data Collection

**Figure 3:**
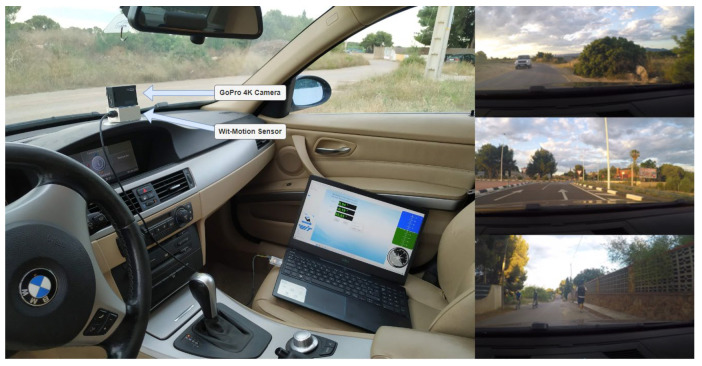
Sensor, camera and laptop installation for real-time sample collection.

The first step was to obtain the actual accelerations of a car under different circumstances. For this purpose, a 10 axis high-precision inertial navigation sensor (Wit-Motion, Shenzhen, China) was used. This sensor, which can be seen in Figure 3, records accelerations (X,Y,Z), angular velocities (rotX, rotY, rotZ), angles (X, Y, Z) and global positioning system (GPS) position. The selected sensor has error margins of less than 0.05 deg, 0.05 deg/s and 0.005 g, which is considered sufficient accuracy for the tests to be performed.

The sensor has several modes of operation. We selected the mode in which real-time data is stored in a .dat file, each time-stamped, at a frequency of 100 Hz. After calibration of the sensor in all axes and measurements, initial tests were performed to ensure that the data obtained were reliable, and the sensor was installed in the central area of the dashboard of a 225 hp BMW 325Xi AUT so that the data obtained could serve as a reference for characterizing the movement of the vehicle.

Regarding the location of the sensor, this is not a trivial matter. The ideal location is the center of mass of the car, but this is somewhat difficult to execute for two reasons. On the one hand, the center of mass is usually inside the vehicle; so, for the installation of the sensor, it would be necessary to disassemble part of the dashboard. On the other hand, car manufacturers do not provide information on the exact location of the center of mass. The first of these handicaps can be avoided by installing it in any other location and making a transformation to the coordinates of the center of mass, but for this it is necessary to know where that is, which leads to the second handicap. Given this inconvenience, the alternative is to record the accelerations suffered by the user and, for this, it is convenient to place the sensor as close as possible to the user’s head, so the easiest option would be to install it on the driver’s headrest. The problem with this installation is that it is not safe to place hardware behind the driver’s head, the seat is equipped with shock absorbers and the driver could hit the headrest, introducing noise into the measurements. Therefore, it was decided to install the sensor on the vehicle’s dashboard, where there is no seat damping and the location is close in height to that of the vehicle’s driver.

In order to temporarily synchronize the data recorded by the sensor with what is happening at each moment during driving, a GoPro camera (San Mateo, CA, USA) installed to record the entire route. For each small event that occurred (going over a speed bump, changing the type of asphalt, crushing some sand from the roadside with a wheel, etc.) the author described it verbally to record it in the video. Figure 3 shows the installation on the car and some snapshots of the videos recorded with the camera.

Several hours of recordings (data and video) were obtained on three types of road surface (gravel, asphalt in poor condition, and asphalt in good condition), different types of roads (urbanization and highway) and with very different events.

**Figure 4:**
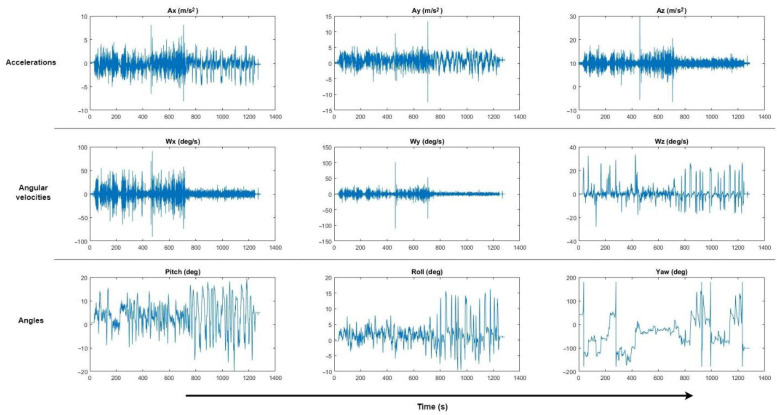
Data collected by the sensor.

Figure 4 shows the appearance of the input data collected from the sensor (accelerations, angular velocities and angles). As can be seen, the bulk of the data corresponds to the vibration produced when driving as a function of speed and the type of road surface, but there are many accelerations that correspond precisely to the events to be evaluated.

### 2.2. DOF Motion Platform Simulation

With the data recorded, the next step was to observe how a motion platform behaves with the input data recorded by the sensor in the performed tests. For this purpose, instead of using a real 6DOF motion platform, which would considerably increase the cost of this study, while making it difficult to measure the accelerations obtained, the platform simulator described in [35] was used.

This simulator has a behavior very similar to that of a real platform, but it is based on numerical calculations performed in real-time by a computer. Thus, given the same inputs as a real platform, the simulator generates similar output signals, which are to be evaluated precisely.

This software can be configured by means of XML files where the physical model of the real platform is fully described (type of motors and physical characteristics, position, mobile and fixed base sizes, motor acceleration ramps, mechanical retracts, etc.). In addition, it is also possible to configure the input data source (in our case, the files with the data collected by the sensors), the simulation frequency (step), the duration of the test depending on the amount of input data available and where the simulation output data should be collected (in our case in separate .dat files for each of the DOFs).

**Figure 5:**
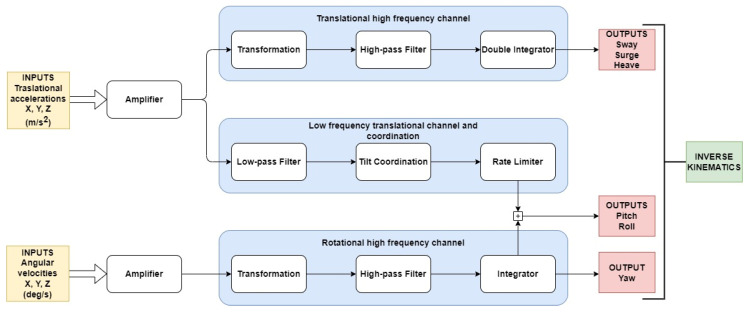
Schematic diagram of a classical washout operation.

The simulated platform was configured to behave as a standard 6DOFs motion platform, designed to support 500 Kg, with motion limits of ±0.15 m for longitudinal (sway, surge and heave) and ±20 deg for rotational (pitch, roll, yaw) motion. Regarding the washout used, in order to try to objectify the study as much as possible, we chose to use the classical washout, based on the initial work of NASA [34] and modified and published by [33]. This is the most widely used washout algorithm at present, despite the time that has passed since it was developed. Figure 5 shows, schematically, the data flow that occurs in the washout.

This algorithm consists of dividing the inputs into three main channels: two for the high-frequency inputs (translational and rotational), which we must always try to reproduce, taking into account the physical limits of the platform, and another one for the low-frequency inputs, which, at the same time, converts part of the low-frequency accelerations in the platform’s inclinations (pitch and roll).

Each of the steps within these channels consists of a series of configurations, which, in our simulator, can be modified within another XML file, thus allowing the desired adjustments to be made depending on the inputs. The final configuration used for the test was the same as in [35] in the T3R3 model.

In order to ensure the correct configuration of the software (washout filters + kinematics + virtual mathematical platform), known signals were introduced, individually, in each of the input channels (Ax, Ay, Az, Wx, Wy, Wz), leaving the rest at 0. At this point it is important to note that the inputs to the algorithm require a small calculation to remove gravity. This is what is known as specific force.

**Figure 6:**
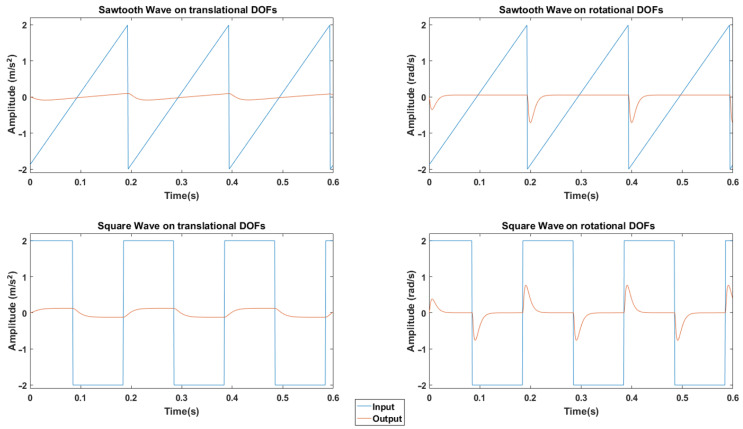
Response of the platform simulation software to well-known inputs.

Specifically, a square and a sawtooth signal, both with a frequency of 5 Hz and amplitude of 2, were introduced to observe the outputs. These signals were introduced individually in each of the axes, observing that the result in the translational channel was the same in all three axes, and the same occurred in the rotational channel. The results obtained, which can be seen in Figure 6, show the behavior of the three translational and three rotational axes in the presence of these well-known signals.

**Figure 7:**
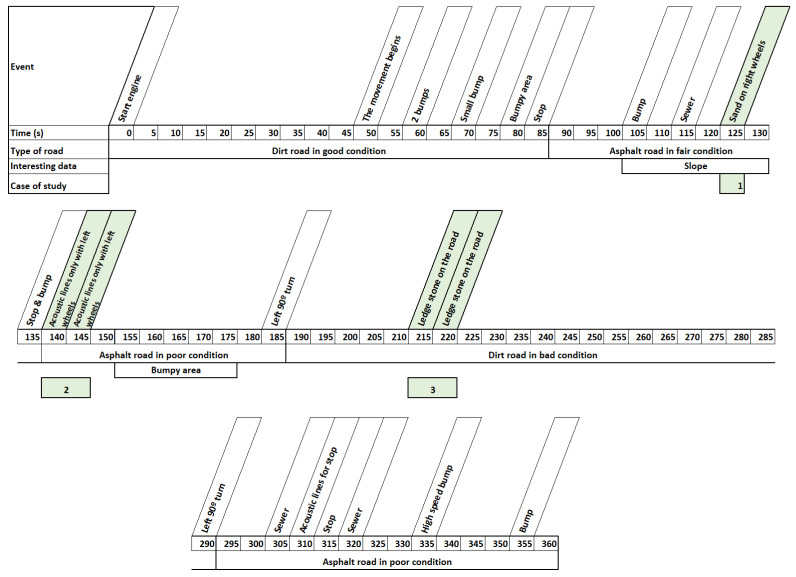
Timeline of the most important events that occurred on a data collection trip.

Once the operation of the motion platform simulator was verified, the data recorded in Section 2.1 were introduced, comparing the input waveforms with the output waveforms. The objective of the analysis was to observe small details that may be lost due to the use of washout filters. To evaluate this, a timeline of the events was made. The timeline, which can be seen in detail in Figure 7, shows the type of road being traveled and the most significant events that were collected.

## 3. Results

The obtained results are based on the comparison of the input data to the software, collected from the real vehicle through the sensor, with the output data from the motion platform simulator. The usual practice in this type of evaluation is to filter the input data obtained from the sensor so that the graphs are “cleaner”. In this case, we preferred not to do so, to avoid losing data and to clearly observe the washout filters’ response and the motion platform’s behavior.

In this case study, instead of numerically observing the inputs concerning the outputs, an analysis is made by comparing the shapes of the signals. As can be seen in the following graphs, in general, the shape of the washout output signal corresponds, with a minimum delay, to the shape of the input signal. For example, the type of road traveled (asphalt in good/bad condition, dirt, etc.) is clearly reflected.

**Figure 8:**
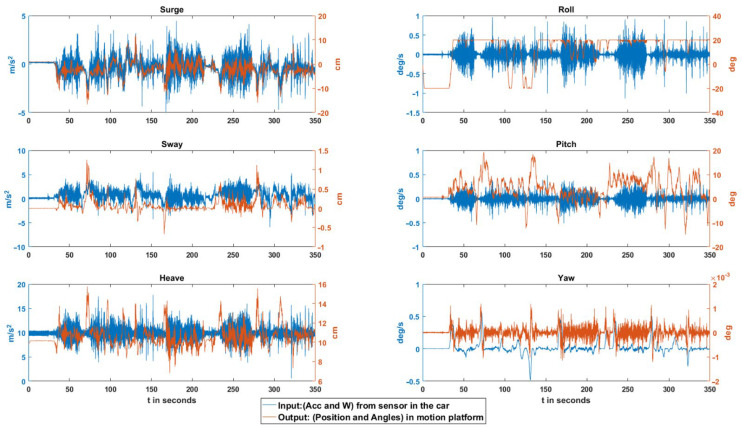
Input/Output results in the different DOFs.

An exhaustive analysis of all the events shown in Figure 8 demonstrates that most of them are adequately reflected in the different filter outputs. However, three are detected to not reflect the input. Specifically, these are road exits in which the right wheels circulated for a few seconds in a sandy area (event 1), sound bands to reduce speed due to the proximity of a yield sign taken only by the left wheels (event 2) and a stone of considerable size on a dirt road (event 3). These events occurred at 125, 140 and 215 s, respectively. The following is a detailed analysis of each of them:

### 3.1. Event 1

**Figure 9:**
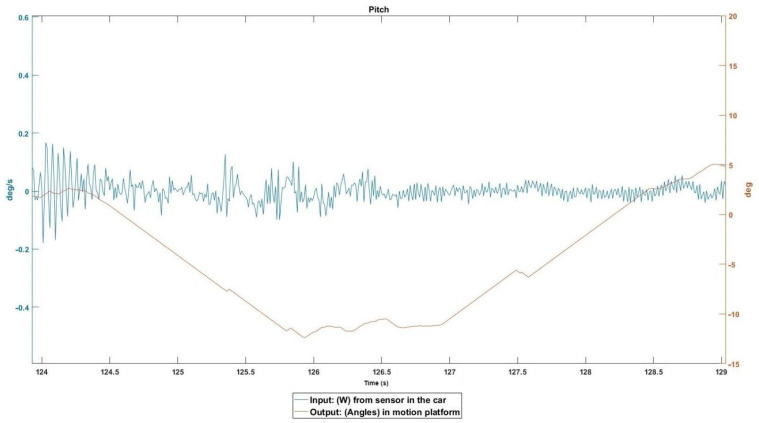
Event 1 details.

The right wheels left the road and drove for 10 s on a smooth sandy surface at about 60 km/h. In Figure 9, the output graph should show a certain “smoothness” but the opposite is true. With “smooth” input, the pitch is triggered because the car was accelerating at that moment, so the tilt coordination adder tells the pitch to simulate acceleration in Y, generating the opposite result.

### 3.2. Event 2

**Figure 10:**
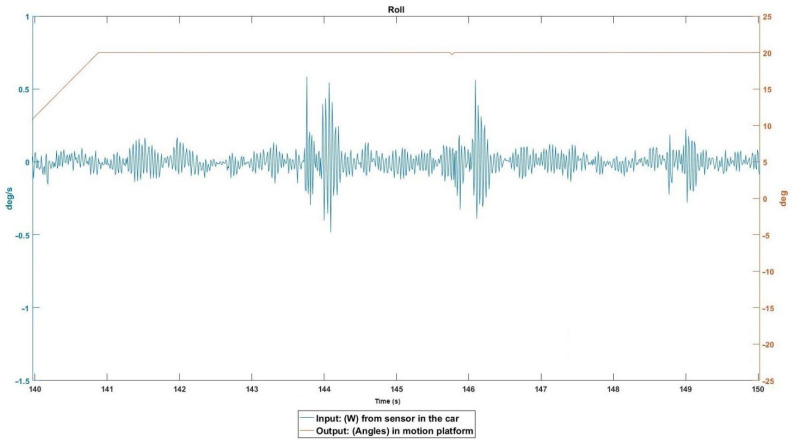
Event 2 details.

The left wheels passed over soundtracks to reduce the speed due to a yield. Figure 10 shows the three soundtracks at the input in angular velocity on the X-axis, but the roll output, which should be in charge of reproducing this, was saturated, so the information on the soundtracks is completely lost. This same (or similar) behavior is observed in the rest of the DOFs. None of them reflect the soundtracks.

### 3.3. Event 3

**Figure 11:**
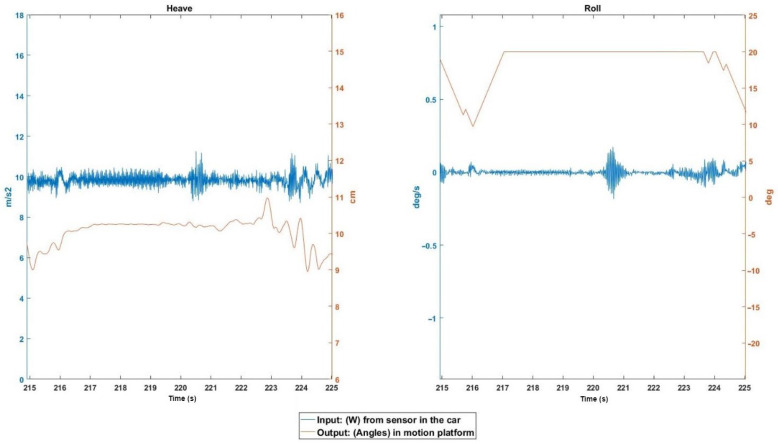
Event 3 details.

Driving on a dirt road, the vehicle passed over a protruding rock at about 20 km/h. In Figure 11, in the AccZ and Wx inputs (for having passed over it with a road), the passage over the rock can be seen at 220.5 s. As can be seen in the outputs of the platform, neither the heave nor the roll reflect the obstacle at all, and therefore it is not appreciated by the user.

## 4. Discussion

Scientific evidence demonstrates the utilities that simulators provide as educational and driver training tools, as well as the importance of the developed systems being realistic for the user [36]. Therefore, the present work has tried to characterize the movements of a vehicle in a typical driving environment, driving on different types of roads and at a wide variety of speeds. Video images have been used to record data for sequencing and classifying the different events that occur during driving. 

These data, based on vehicle accelerations and angular velocities, have been fed into a motion platform simulator, whose correct operation has been checked by inputting and analyzing the output of well-known signals; output data in terms of positions of each DOF were obtained. From the analyses performed, it can be observed that, most of the time, the washout filters and the motion platforms have an expected behavior, transmitting, within the constructive possibilities of each platform, the accelerations that a user in a simulator expects to perceive. 

However, three specific events have been observed in which the output information, i.e., the motion that is transmitted to the user of a simulator with a motion platform, does not correspond to the input information that occurred in real driving. This implies that, in these three specific events, vestibular information is lost. These data do not differ from other investigations since multiple pieces of evidence indicate that simulators may have certain limitations in the reproduction of the situations or scenes presented, derived from the characteristics of these devices [29,30,31,32,33,34,35,36,37,38,39]. The loss of vestibular information may be due to several causes. First, the physical/constructive limits of the motion platform are very restricted. Second, priorities are set on the DOFs that cause some of the information (lower priority DOFs) to be lost. Finally, the washout filters do not allow certain accelerations to pass to the platform.

In the present case, we have found that there are three specific events that the motion platform does not transmit to the user, losing part of the information that the user should receive. With a different configuration of the washout filters or with another motion platform, these movements could possibly have been reproduced, but there will always be some that are lost and, therefore, not perceived by the user.

There is extensive literature on the subject [40,41,42,43], in which attempts have been made to increase the user’s perception by applying scaling to the different channels that make up the washout filters. In general, it is concluded that the application of scaling to certain washout channels, always within certain limits, is usually positive [44] since it increases the user’s perceptual level, but there is no consensus on how to apply them. 

Analyzing the literature, it can be observed that many authors state that the vestibular perception of vehicle users directly affects decision-making in the driving process, both in the reaction times and in the routes, among others. Therefore, it can be concluded that the users of a simulator with a motion platform do not perceive certain events that are considered important in driving through their vestibular system. Thus, the loss of information on elements such as the type of ground on which one is driving or the non-perception of soundtracks that reduce speed will directly affect the decisions made by the user in the simulation. Therefore, this could affect the quality of learning, and even limit or distort the feedback that the instructor could give. 

Thus, the user’s perception of what is happening, whether in real driving or a simulation, has been shown [45] to be key to reducing reaction times, deviations in the road trajectory or increasing the level of alertness, among others. Evidence shows that an unrealistic risk perception is one of the main risk factors in road accidents [46]. Low levels of risk perception are related to the overestimation of driving abilities and, consequently, to irresponsible behaviors such as alcohol consumption while driving, distractions, or traveling at a higher speed than allowed [47]. 

In this sense, to achieve a correct risk perception, it is necessary that the user has an adequate awareness, as well as a perception of the road conditions adjusted to reality [48]. 

This aspect could be enhanced and trained through the use of simulators. In certain countries, there is a very strict regulation when it comes to obtaining a driving license with the use of simulators. These are the UK [49], the Netherlands [50], Singapore [51] and Finland [52], where driving simulators are widely used for training new drivers and even for obtaining a driver’s license, for which they must analytically pass certain tests. If the inputs perceived by the user are not adequate, will the user’s reactions and, therefore, the result obtained from these simulations be adequate?

According to Kemeny and Panerai [53], having vestibular information (provided by the motion platform) in driving simulators decreases the reaction times to the events occurring in the simulation. On the other hand, Reymond et al. [54] affirm that drivers on curved roads, in order to establish their car control strategies, make use, not only of the visual system, but also of the vestibular system.

In the case of training simulators, such as those discussed for driving schools, in the authors’ opinion, it is of vital importance that the user perceives the accelerations of any of the events that have been shown to be lost. Otherwise, their perception and reaction to what is happening in the simulation will be distorted. In addition, in order to properly evaluate the users, they must have a true perception of all road and vehicle conditions. 

The case study conducted is not without limitations. First, since the data collected by the sensors and the data extracted by the MCA algorithm are not numerically comparable, the study was based on observing the shape of the graphs, independently, of each DOF in the presence of certain inputs. From the information analyzed and based on the experience of the research team and bibliographic evidence, the conclusions shown in the following chapter have been drawn.

Secondly, the reader may think that this study could have been carried out by comparing the data recorded by various vehicles. However, since it is clear that the scientific community assures that it is impossible to reproduce 100% of the accelerations that occur in a real vehicle using a motion platform, the only thing that would be achieved is to detect other different events that would produce losses. The only impact this would have is to ratify the results presented here. 

## 5. Conclusions

Given the importance of the human factor in traffic accidents, preventive measures should be established to help reduce driver errors, violations, and, consequently, accident rates. In this sense, developing valid and effective training and education tools is fundamental for the driver’s correct learning and awareness.

Technological devices can be very useful for this purpose, especially those related to virtual reality and driving simulators [55]. However, these tools are constantly changing, adapting to the needs of users, and increasing their level of realism. Primarily, the perceptual aspects of simulators need to be improved. In particular, evidence indicates that motion sensation is very important in achieving realistic driving sensations for the user. 

Thus, the present research indicates that motion platforms transmit, according to their capabilities, the accelerations that a user in a simulator expects to perceive. However, it has been shown that there are small events that are not adequately reproduced by the motion platforms. There are few options to guarantee that a motion platform will always reproduce certain accelerations that are considered important, since this will depend on how the motion cueing algorithms (MCA) are placed and how the platform is physically positioned or what happens in the simulation. In this sense this work introduces evidence that allows this extreme scenario to be mitigated. These findings are interesting from the perspective of using simulators to investigate one’s own behavior, especially concerning the differential decision-making process [56].

As future work, the authors propose a possible solution to ensure that the events that are considered important are reproduced according to the use made of each simulator. The solution will lie with the introduction of small heuristics that go through a parallel channel to the classical washout algorithm and ensure, by preparing the position of the actuators, that certain events are perceived as accelerations by the user.

## Figures and Tables

**Table 1:** Advantages vs disadvantages of the research.

Advantages
It allows the qualitative detection of certain driving elements that are not reflected in the motion platform
The impact of the sensations lost as a result of the training with a driving simulator was studied.
A possible solution is proposed so that these motion cues that are lost can be transferred to the user
Disadvantages
Being a qualitative analysis, there are no exact measures of “how much” is lost
Repeating the test with the exact same conditions is very difficult (temperature, speed, traffic, road situation, etc.)

## Data Availability

The study data can be accessible upon reasonable request to the corresponding author.

## References

[1] Azami-Aghdash S., Aghaei M.H., Sadeghi-Bazarghani H. (2018). Epidemiology of road traffic injuries among elderly people; A systematic review and meta-analysis. Bull. Emerg. Trauma.

[2] McIlroy R.C., Plant K.A., Hoque M.S., Wu J., Kokwaro G.O., Nam V.H., Stanton N.A. (2019). Who is responsible for global road safety? A cross-cultural comparison of Actor Maps. Accid. Anal. Prev..

[3] Cociu S. (2020). Environmental risk factors related to road traffic crashes. Arta Med..

[4] Allerton D. (2009). Principles of Flight Simulation.

[5] Rolfe J.M., Staples K.J. (1986). Flight Simulation.

[6] Chou C.T., Fu L.C. Ships on Real-time Rendering Dynamic Ocean Applied in 6DOF Platform Motion Simulator. Proceedings of the CACS International Conference 2007.

[7] Casas S., Rueda S., Riera J.V., Fernández M. On the Real-Time Physics Simulation of a Speed-Boat Motion. Proceedings of the International Conference on Computer Graphics Theory and Applications (GRAPP 2012).

[8] Slob J.J. (2008). State-of-the-Art Driving Simulators: A Literature Survey.

[9] Thöndel E. Design and Optimisation of a Motion Cueing Algorithm for a Truck Simulator. Proceedings of the European Simulation and Modelling Conference 2012 EUROSIS.

[10] Xu Z., Han S.H. (2012). Submarine Behavior Simulation based on 4-DOF Motion Platform and Stereoscopic Multi-Channel Visualization. Trans. Soc. CAD/CAM Eng. Soc. Comput. Des. Eng..

[11] Romoser M.R., Fisher D.L. (2009). The effect of active versus passive training strategies on improving older drivers’ scanning in intersections. Hum. Factors.

[12] Casutt G., Theill N., Martin M., Keller M., Jäncke L. (2014). The drive-wise project: Driving simulator training increases real driving performance in healthy older drivers. Front. Aging Neurosci..

[13] Schreier D.R., Banks C., Mathis J. (2018). Driving simulators in the clinical assessment of fitness to drive in sleepy individuals: A systematic review. Sleep Med. Rev..

[14] Ekanayake H.B., Backlund P., Ziemke T., Ramberg R., Hewagamage K.P., Lebram M. (2013). Comparing Expert Driving Behavior in Real World and Simulator Contexts. Int. J. Comput. Games Technol..

[15] De Winter J.C.F., van Leeuwen P.M., Happee R. Advantages and Disadvantages of Driving Simulators: A Discussion. Proceedings of the Measuring Behavior 2012.

[16] Käppler W.D. Views on the role of simulation in driver training. Proceedings of the 12th European Annual Conference on Human Decision Making and Manual Control.

[17] Lee J.D. Simulator fidelity: How low can you go?. Proceedings of Measuring Behavior 2012.

[18] Reed M.P., Green P.A. (1999). Comparison of driving performance on-road and in a low-cost simulator using a concurrent telephone dialling task. Ergonomics.

[19] Vlakveld W.P. The Use of Simulators in Basic Driver Training.

[20] Berg U., Wojke P., Zöbel D. (2008). Driver training simulator for backing up commercial vehicles with trailers. J. Mech. Syst. Transp. Logist..

[21] Zeeb E. (2010). Daimler’s new full-scale, high-dynamic driving simulator—A technical overview. Trends in Driving Simulation Design and Experiments.

[22] Dongsu W., Hongbin G. (2007). Adaptive Sliding Control of 6DOF Flight Simulator Motion Platform. Chin. J. Aeronaut..

[23] Carsten O., Jamson A.H. (2011). Driving simulators as research tools in traffic psychology. Handbook of Traffic Psychology.

[24] Beghi A., Bruschetta M., Maran F. (2012). A real time implementation of MPC based Motion Cueing strategy for driving simulators. Proceedings of the 2012 IEEE 51st IEEE Conference on Decision and Control (CDC), Maui, HI, USA, 10–13 December 2012.

[25] Miermeister P., Lächele M., Boss R., Masone C., Schenk C., Tesch J., Bülthoff H.H. (2016). The cablerobot simulator large scale motion platform based on cable robot technology. Proceedings of the 2016 IEEE/RSJ International Conference on Intelligent Robots and Systems (IROS), Daejeon, Korea, 9–14 October 2016.

[26] Casas S., Coma I., Riera J.V., Fernández M. On the Characterization of a Speed-boat Motion for Real-time Motion Cueing. Proceedings of the International Conference on Computer Graphics Theory and Applications (GRAPP 2013).

[27] Langåker A., Ngo S. (2021). Investigating Motion Sickness in Racing Simulators using Virtual Reality and a Motion Platform. Master’s Thesis.

[28] Stewart D. (1965). A Platform with 6 Degrees of Freedom. Proc. UK Inst. Mech. Eng..

[29] Hummel S.R., Chassapis C. (1998). Configuration design and optimization of universal joints. Mech. Mach. Theory.

[30] Gosselin C. (1990). Determination of the Workspace of 6-DOF Parallel Manipulators. J. Mech. Des..

[31] Majid M., Huang Z., Yao Y. (2000). Workspace Analysis of a Six-Degrees of Freedom, Three-Prismatic- Prismatic-Spheric-Revolute Parallel Manipulator. Int. J. Adv. Manuf. Technol..

[32] Liu K., Fitzgerald J.M., Lewis F.L. (1993). Kinematic analysis of a Stewart platform manipulator. IEEE Trans. Ind. Electron..

[33] Reid D.L., Nahon M.A. (1985). Flight Simulation Motion-Base Drive Algorithms: Part 1—Developing and Testing the Equations.

[34] Schmidt F.S., Conrad B. (1969). The Calculation of Motion Drive Signals for Piloted Flight Simulators.

[35] Casas S., Alcaraz J.M., Olanda R., Coma I., Fernández M. (2014). Towards an extensible simulator of real motion platforms. Simul. Model. Pract. Theory.

[36] Asadi H., Lim C.P., Mohamed S., Nahavandi D., Nahavandi S. (2019). Increasing motion fidelity in driving simulators using a fuzzy-based washout filter. IEEE Trans. Intell. Veh..

[37] Konstantopoulos P., Chapman P., Crundall D. (2010). Driver’s visual attention as a function of driving experience and visibility. Using a driving simulator to explore drivers’ eye movements in day, night and rain driving. Accid. Anal. Prev..

[38] Olstam J.J., Lundgren J., Adlers M., Matstoms P. (2008). A framework for simulation of surrounding vehicles in driving simulators. ACM Trans. Modeling Comput. Simul. TOMACS.

[39] Fisher D.L., Pollatsek A., Horrey W.J. (2011). Eye behaviors: How Driving Simulators Can Expand Their Role in Science and Engineering.

[40] Wynne R.A., Beanland V., Salmon P.M. (2019). Systematic review of driving simulator validation studies. Saf. Sci..

[41] Van Leeuwen T.D., Cleij D., Pool D.M., Mulder M., Bülthoff H.H. (2019). Time-varying perceived motion mismatch due to motion scaling in curve driving simulation. Transp. Res. Part F Traffic Psychol. Behav..

[42] Chen S.H., Fu L.C. (2010). An optimal washout filter design for a motion platform with senseless and angular scaling maneuvers. Proceedings of the 2010 American Control Conference, Baltimore, MD, USA, 30 June–2 July 2010.

[43] Asadi H., Mohammadi A., Mohamed S., Qazani M.R.C., Lim C.P., Khosravi A., Nahavandi S. (2019). A model predictive control-based motion cueing algorithm using an optimized nonlinear scaling for driving simulators. Proceedings of the 2019 IEEE International Conference on Systems, Man and Cybernetics (SMC), Bari, Italy, 6–9 October 2019.

[44] Berthoz A., Bles W., Bülthoff H.H., Grácio B.C., Feenstra P., Filliard N., Wentink M. (2013). Motion scaling for high-performance driving simulators. IEEE Trans. Hum. Mach. Syst..

[45] Zöller I., Abendroth B., Bruder R. (2019). Driver behaviour validity in driving simulators–Analysis of the moment of initiation of braking at urban intersections. Transp. Res. Part F Traffic Psychol. Behav..

[46] Li Y., Zheng Y., Wang J., Kodaka K., Li K. (2018). Crash probability estimation via quantifying driver hazard perception. Accid. Anal. Prev..

[47] MacLeod K.E., Karriker-Jaffe K.J., Ragland D.R., Satariano W.A., Kelley-Baker T., Lacey J.H. (2015). Acceptance of drinking and driving and alcohol-involved driving crashes in California. Accid. Anal. Prev..

[48] Horswill M.S., Hill A., Wetton M. (2015). Can a video-based hazard perception test used for driver licensing predict crash involvement?. Accid. Anal. Prev..

[49] Straus S.H. (2005). New, Improved, Comprehensive, and Automated Driver’s License Test and Vision Screening System.

[50] Sætren G.B., Pedersen P.A., Robertsen R., Haukeberg P., Rasmussen M., Lindheim C. (2018). Simulator training in driver education—Potential gains and challenges. Safety and Reliability–Safe Societies in a Changing World.

[51] Upahita D.P., Wong Y.D., Lum K.M. (2018). Effect of driving inactivity on driver’s lateral positioning control: A driving simulator study. Transp. Res. Part F Traffic Psychol. Behav..

[52] Bro T., Lindblom B. (2018). Strain out a gnat and swallow a camel?—Vision and driving in the Nordic countries. Acta Ophthalmol..

[53] Kemeny A., Panerai F. (2003). Evaluating perception in driving simulation experiments. Trends Cogn. Sci..

[54] Reymond G., Droulez J., Kemeny A. (2002). Visuovestibular perception of self-motion modeled as a dynamic optimization process. Biol. Cybern..

[55] Riera J.V., Casas S., Fernández M., Alonso F., Useche S.A. (2021). Development of a Hybrid Method to Generate Gravito-Inertial Cues for Motion Platforms in Highly Immersive Environments. Sensors.

[56] Machado-León J.L., de Oña J., de Oña R., Eboli L., Mazzulla G. (2016). Socio-economic and driving experience factors affecting drivers’ perceptions of traffic crash risk. Transp. Res. Part F Traffic Psychol. Behav..

